# Monsters

**Published:** 1921-01-15

**Authors:** 


*To the Editor of* The Hospital.

### "Monsters."

[We have received the following letter from a correspondent, and print it in full, with one or two small omissions of names, etc.]

*To the Editor of* The Hospital.

Sir,—The conduct of hospital secretaries as regards applicants for resident appointments at their institutions amounts to a scandal.

Advertisements are inserted in the medical Press inviting applicants (and copies of testimonials to be enclosed) for resident appointments at certain hospitals. When applications are sent and copies of testimonials enclosed, these monsters (hospital secretaries) are so absolutely lacking in the common civilities of life that they do not even take the trouble to acknowledge the applications which they themselves have invited! while others who do acknowledge them have the indecency to do so on a postcard and in red ink (favourable or otherwise to the applicant). This complaint is nothing new against secretaries of hospitals—see The Hospital for May 31, 1919, and *British Medical Journal* for December 1920. But it's time it were put an end to. We applied and sent three copies of testimonials a fortnight before Christmas for a resident post to two secretaries. Up to date I have not had a word from either in any way acknowledging my application and testimonials. Secretaries of this type can be of no credit to any institution. We cannot help wondering whether certain hospital secretaries have the intelligence to realise the great damage they do to the hospital by their mean and despicable conduct? We have held a number of resident appointments and have the best of testimonials. We are not in the least concerned about not securing either of these posts, but we do object to the gratuitous insult and insolence.

By waiting until now I think you will agree that I have given these secretaries plenty of time to reply if they had any intention of doing so. I want my copies of testimonials back, that's what worries me, and if I can't get them returned without forwarding postage, then that fact should have been inserted with the advertisement. I make no excuse for writing to you in this matter. Such conduct on the part of hospital secretaries would be denounced by every thinking man and woman the world over as most unworthy of the character and nature of the institution they represent!—Yours faithfully,

